# Guiding Principles for Science-Based Food Classification Systems Focused on Processing and Formulation^☆^^☆☆^

**DOI:** 10.1016/j.advnut.2025.100577

**Published:** 2026-01-19

**Authors:** Jodi T Bernstein, Andrew W Brown, Britt Burton-Freeman, Mario Estevez, Julie M Hess, Patrice A Hubert, Marie E Latulippe

**Affiliations:** 1 Jodi Bernstein Medical Writing, Toronto, ON, Canada; 2 Department of Biostatistics, University of Arkansas for Medical Sciences and Arkansas Children’s Research Institute, Little Rock, AR, United States; 3 Department of Food Science and Nutrition, Director, Center for Nutrition Research, Illinois Institute of Technology, Chicago, IL, United States; 4 Research Institute in Meat and Meat Products, Veterinary School, University of Extremadura, Cáceres, Spain; 5 USDA, Agricultural Research Service, Grand Forks Human Nutrition Research Center, Grand Forks, ND, United States; 6 Monell Chemical Senses Center, Philadelphia, PA, United States; 7 Institute for the Advancement of Food and Nutrition Sciences, Washington, DC, United States

**Keywords:** food classification systems, food processing, food formulation, ultraprocessed foods, highly processed foods, guiding principles, scientific principles, nutrition policy

## Abstract

Food classification systems that focus on food formulation & processing classification (FF&PC systems) have gained traction in research and dietary policies. Yet, their utility and scientific foundations are debated. To address criticisms and identify paths forward, the Institute for the Advancement of Food and Nutrition Sciences convened a Working Group comprising government, industry, and academic scientists to conceptualize a project to meet the needs of the scientific community in addressing recurring concerns about FF&PC systems in the literature. Born from this, an independent public sector Writing Team with expertise in food science, nutrition research and methodology, dietary guidance, and sensory science led the development of guiding principles for researchers to consider when developing, refining, and applying FF&PC systems for public health. The principles emphasize the need for transparent documentation; distinguishable versioning; strong, and ideally causal, evidence of a putative effect of specific processing steps and formulation components with health-related endpoints; evolution over time in response to scientific advances and changes to the food supply; and consideration of current scientific consensus, validation contexts, and the probative value of research questions and new FF&PC systems. These principles are intended to provide a shared foundation and standardized approach to guide researchers in this area without endorsing or advocating for the creation or use of FF&PC systems. These aspirational principles can be used to identify future research priorities and areas for investment while also providing guidance on cautionary action in the absence of complete data. Adherence to these principles is also intended to limit the continued expenditure of resources critiquing or defending new FF&PC systems. These principles can serve as a foundation to support impactful research on FF&PC systems and, through this, public health policy.

**Statement of Significance**

Developed through a multistakeholder collaboration, this perspective proposes 9 principles to guide the development, refinement, and application of food classification systems focused on processing and formulation. It offers a framework for aligning systems with scientific standards and public health policy goals while allowing flexibility for researchers to tailor system design to specific use cases.

## Introduction

Food classification systems have been proposed and applied through religious doctrine, social habits, and policy guidance for millennia (e.g. halal, kosher) [1]. In the last 15 years, various food classification systems have emerged with the intention of using more modern, systematic, or scientific definitions, with several focusing on processing and formulation [[2], [3], [4], [5], [6], [7], [8]]. Briefly, food processing refers to the transformation of agricultural products into food ingredients or finished food products using various methods and technologies [9], and formulation involves selecting and combining specific ingredients used to create a final product [10,11]. Food processing has been described as “the use of methods and techniques involving equipment, energy, and tools to transform agricultural products such as grains, meats, vegetables, fruits, and milk into food ingredients or finished food products” [9]. Formulation refers to the selection and proportioning of ingredients (e.g. macronutrients, micronutrients, additives, and other components) used to create a final product [10,11].

**FIGURE 1:**
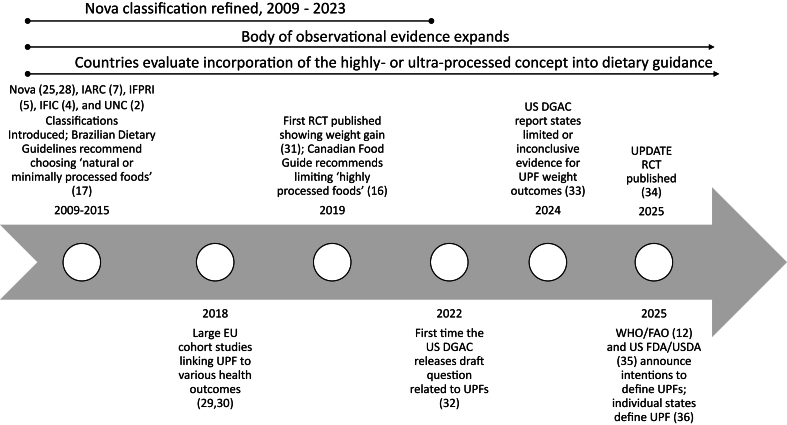
Highlighted activities related to food formulation and processing classification systems supporting the value of universal guiding principles. EU, European Union; FDA, Food and Drug Administration; IARC, International Agency for Research on Cancer; IFIC, International Food Information Council; IFPRI, International Food Policy Research Institute; RCT, randomized controlled trials; UNC, University North Carolina; UPDATE, “Ultra-processed vs. minimally processed diets following UK dietary guidance on health outcomes” trial; UPF/UPFs, ultra-processed food(s); US DGAC, United States Dietary Guidelines Advisory Committee.

Food classification systems that categorize foods based on processing and formulation [herein “food formulation and processing classification (FF&PC) systems”] have gained traction in both epidemiological and interventional research and dietary policies and recommendations [[12], [13], [14], [15], [16], [17], [18], [19], [20], [21], [22], [23]]. Most FF&PC systems describe foods along a continuum according to the extent, purpose, or degree of processing, ranging from unprocessed or minimally processed to highly or ultraprocessed [A recent definition of “ultra-processed foods” according to the Nova classification system is “(i)ndustrially manufactured food products made up of several ingredients (formulations) including sugar, oils, fats and salt (generally in combination and in higher amounts than in processed foods) and food substances of no or rare culinary use (such as high-fructose corn syrup, hydrogenated oils, modified starches and protein isolates)…]” [6]. The definitions of highly or ultra-processed foods vary by classification system but commonly reference industrial preparation, multiple ingredients, readily edible formats requiring little to no domestic preparation, loss of resemblance to the original plant or animal source, and/or high levels of added sugars, fats, or sodium [2,5,7,8,24], with variations in category names and criteria [[2], [3], [4], [5], [6], [7], [8],^25^,^26^]. The Nova classification system [3] was one of the earliest modern FF&PC systems introduced and is a widely used system in literature today [27]. In addition to Nova, several other FF&PC systems have been (and continue to be) developed. Dietary guidance related to FF&PC systems tends to recommend limiting or avoiding intakes of foods that are highly or ultra-processed or to give preference to unprocessed or minimally processed foods [[15], [16], [17], [18], [19], [20], [21], [22], [23]]. Highlights of system development, research progress, and policy developments over the past 20 y related to FF&PC are presented in Figure 1 [2,4,5,7,12,16,17,25,[28], [29], [30], [31], [32], [33], [34], [35], [36]].

## Rationale

Despite their growing use, the utility and scientific basis of such FF&PC systems have been called into question and extensively debated in the literature [10,11,13,26,[37], [38], [39], [40], [41], [42], [43], [44], [45], [46], [47], [48], [49], [50], [51], [52], [53]]. Although reviewing and summarizing all critiques are beyond the scope of the present manuscript, an abbreviated list of concerns with select references is presented in Box 1.

Box 1 **Recurring concerns about food classification systems focused on processing and formulation∗**

• Inconsistency within and between food classification systems [13,26,[37], [38], [39], [40], [41]].
• Ambiguity in definitions, purpose, and methodology [13,26,37,38,[40], [41], [42], [43], [44], [45], [46], [47], [48], [49]].
• Inadequate consideration of biological mechanisms and relationships with health-related outcomes [37,40,46,49].
• Limited availability and quality of evidence [13,26,37,40,42,46,47,[50], [51], [52]].
• Insufficient separation of food formulation and processing operations [10,11,13,38,43,47].
• Misalignment between food classification systems and prevailing nutrition guidance and science [11,13,26,38,40,[44], [45], [46],^49^,^52^].
• Potential for overgeneralization and application of systems [39].
• The probative value of creating and using food classification systems over and above other nutrition considerations [13,40,48,49,52,53].

∗This is not an exhaustive list of all concerns, and the references cited are not intended to represent the entirety of the existing literature.

Alt-text: Box 1

To reflect on the concerns and identify paths forward, in 2023, the Institute for the Advancement of Food and Nutrition Sciences (IAFNS) convened a cross-stakeholder Food Classification Workshop to evaluate existing FF&PC systems, including their scientific basis, validity, and utility in researching food-health relationships and informing policy decisions. This collaborative effort resulted in a 2024 perspective piece authored by 18 food and nutrition scientists, highlighting a shared recognition that advancing food classification in a health-relevant direction depends on establishing clear scientific criteria and methodological principles [40]. These recommendations provided the foundational premise for the work presented herein.

This perspective presents 9 guiding principles for the scientific community to consider when developing, refining, and applying FF&PC systems, with the intention of supporting research that informs effective health policies. These principles do not endorse or advocate for the creation of new FF&PC systems or the use of existing FF&PC systems in research. Instead, these principles provide a shared foundation and a standardized approach to guide researchers from diverse disciplines who choose to pursue this line of inquiry. Adherence to these principles is also intended to reduce the likelihood of future resources being diverted toward critiquing, defending, or responding to skepticism about the merits of new FF&PC systems.

## Methods

Additional details on the process for principles development are available in Supplementary Materials. Briefly, IAFNS convened a Working Group of scientists from government, industry, and academia. The group aimed to build consensus among food and nutrition stakeholders on the guidance and evidence requirements for classifying foods based on processing and formulation to inform research that can support public health. The Working Group identified and assembled an independent Writing Team to draft a set of guiding principles drawing on expert collaboration, a targeted literature review, and multidisciplinary stakeholder feedback, with the direction that no more than 10 principles should be developed. The Writing Team met in January 2025 to define the process, discuss the literature, and share feedback. Additionally, the Writing Team gathered during a workshop hosted by IAFNS in April 2025. Workshop participants included nutrition scientists, food technologists, toxicologists, public health experts, and regulatory professionals; their inputs were used to refine the principles.

The Writing Team developed 9 principles (Box 2) to guide researchers in developing and refining FF&PC systems that incorporate processing and formulation in transparent, robust, and reproducible ways suitable for research intended to inform policy and support public health. The Writing Team adopted the definition of a principle as “a fundamental truth or proposition on which others depend; a general statement or tenet forming the (or a) basis of a system of belief […]; a primary assumption forming the basis of a chain of reasoning” [55]. Each principle was designed to address specific issues with FF&PC systems highlighted in the literature [10,11,13,26,[37], [38], [39], [40], [41], [42], [43], [44], [45], [46], [47], [48], [49], [50], [51], [52], [53]]. Given the project’s focus on health relevance, other outcomes, recognized as relevant to the overall food system and dietary choices, such as affordability, convenience, availability, sustainability, food waste, and marketing, among others, were considered outside the scope of these principles.

Box 2 **2025 Guiding principles for classifying foods based on processing and formulation**

1. Documentation and definitions that allow for reproducibility, rigor, and transparency should be provided.
2. Properties for which there is evidence of a biological link with a health-related endpoint should be used to differentiate foods.
3. Associations without robust causal evidence should be considered preliminary.
4. The impact that processing steps have on the final composition and structure of the food in terms of a putative effect on a health-related endpoint should be considered.
5. The impact of formulation on the final composition and structure of the food in terms of a putative effect on a health-related endpoint should be considered.
6. Systems should evolve over time to reflect advancements in science and changes in the food supply, with previous versions of a system being distinguishable from updated versions.
7. Current scientific evaluations from scientific bodies with relevant expertise should be consulted for each iteration.
8. The context(s) in which a system was validated should be considered in its application.
9. The probative value of a research question or proposed FF&PC system should be considered before engaging in analysis or development.

Alt-text: Box 2

### Guiding principles for food classification systems focused on processing and formulation

#### Documentation and definitions that allow for reproducibility, rigor, and transparency should be provided

FF&PC systems must be underpinned by rigorous and transparent documentation to enable consistent and appropriate applications. [Reproducibililty, rigor, and transparency are considered as defined in reference [56]. Reproducibility as related to computational reproducibility refers to “obtaining consistent results using the same input data, computational methods, and conditions of analysis.” Rigor is the “strict application of the scientific method to ensure robust and unbiased experimental design.” Aspects of transparency include a scientific report that “makes clear whether the study was exploratory or confirmatory, shares information about what measurements were collected and how the data were prepared, which analyses were planned and which were not, and communicates the level of uncertainty in the result (e.g. through an error bar, sensitivity analysis, or *P* value).”]. A key tenet of science is that results should be repeatable under the same conditions by independent researchers, which is facilitated with comprehensive accompanying documentation; the same applies to food classification systems. This principle emphasizes the importance of clearly documenting, at a minimum, the point of classification (e.g. purchase compared with consumption), all required inputs (e.g. ingredients, processing steps, chemical analyses), relevant food properties (e.g. bioavailability, digestibility), intended outcomes of interest (e.g. cardiometabolic risk), target populations (e.g. adults, specific countries), methods for quantifying intake (e.g. biomarkers, self-report, purchase history), assumptions made (e.g. foods in a given classification group have the same effect on health), foreseeable limitations, and exhaustive definitions of key terms.

The second component of this principle focuses on definitions. Definitions should be objective, measurable, and grounded in scientific consensus. Including a glossary of terms can further support transparency. One common critique of the descriptor “ultra-processed food” is that it lacks clarity and is neither explicitly nor quantitatively defined [37,41,43,48]. Although the introduction of new terms when creating or refining FF&PC systems is not discouraged, this principle holds that it should be accompanied by a clear definition and measurement methods (e.g. “energy density” measured as kilocalories per gram). The use of novel or ambiguous terms (e.g. “natural,” “culinary preparations,” “home cooking,” “whole food,” “normally”) should be scientifically justified and consistently applied [26,37,48]. When reusing existing terms, definitions should be explicitly reiterated with reference to the original source and supported by a defensible scientific rationale. Researchers should avoid repurposing terms that are already defined in other disciplines, which may potentially lead to misinterpretation. For example, ultrafiltration and food matrix are terms with specific meanings in the fields of food science and technology. To guard against this, engaging multidisciplinary expertise spanning nutrition science, toxicology, food science, food engineering, public health, and behavioral science is essential. Terminology should also be clearly distinguished from related but nonequivalent concepts, such as “formulation” compared with “processing.”

This principle emphasizes the importance of clearly documenting methodologies, underlying assumptions, and limitations to ensure appropriate and transparent application of FF&PC systems to enable replication and evaluation of related research that could be used to support decision-making. Given its foundational nature, this principle also serves as an overarching requirement that applies across all other principles presented here.

#### Properties for which there is evidence of a biological link with a health-related endpoint should be used to differentiate foods

This principle ensures FF&PC systems are grounded in properties supported by mechanistic evidence, presumably on the causal pathway between consumption and health outcomes (see principle 3), rather than assumptions or heuristic proxies. The purported mechanisms and their limitations should also be transparently documented (principle 1). Current systems have been criticized for using properties to classify foods based on hypothetical relationships rather than validated links to health outcomes [26]. Using the Nova classification system as an example, category 4 (ultra-processed) foods are identified in part by use of ingredients with similarly “cosmetic functions,” which includes a wide variety of approved food additives with varied chemical compositions, each with its own evidence base (or lack thereof) linking the ingredient to health effects [26].

FF&PC systems should distinguish foods based on properties with empirically testable causal relations with health-related endpoints. At the discretion of the system developers, endpoints may include acute issues (e.g. food safety, pathogen risk), chronic conditions (e.g. obesity, cardiovascular disease, type 2 diabetes), related biomarkers (e.g. hypertension, cholesterol), or intermediary mechanisms (e.g. satiety, postprandial glucose response). Food properties may cover aspects of the whole food (e.g. texture), processing changes (e.g. nutrient loss, by-product formation) (principle 4), or aspects of formulation (e.g. sugar content, added nitrates) (principle 5). The classification of foods provides a valuable basis for subsequent dose–response studies and dietary pattern analyses. The responsibility falls on the developers to proactively justify the inclusion of each food property in the system and produce evidence for each.

Full adherence to this principle depends on advancing our mechanistic understanding of how specific food properties are beneficial or detrimental to health [57]. In the meantime, transparency in documentation (principle 1) can be leveraged to clearly distinguish FF&PC systems that rely on observational evidence rather than evidence produced from experimental study designs where causality can be more confidently inferred, as well as the implications of using such a system for public health decision-making in the absence of complete data.

#### Associations without robust causal evidence should be considered preliminary

This principle builds on principle 2 to specify that in the absence of strong evidence supporting causal relationships, any conclusions should be considered preliminary and explicitly communicated as such.

In nutrition research, much of the literature is observational in design. Although observational studies represent a continuum of designs, most observational nutrition studies are based on dietary intake and association tests that can suggest associations and generate hypotheses, but are limited in their ability to establish causality—a limitation acknowledged in the literature on FF&PC systems [48,51,58]. Randomized controlled trials (RCTs) are considered the reference standard for establishing cause-and-effect relationships, but they are not always feasible, ethical, or well-executed [59]. In the absence of robust causal data from RCTs, other well-conducted study designs can be considered collectively, and can be evaluated using frameworks like the Bradford Hill criteria [60], Grading of Recommendations Assessment, Development and Evaluation (https://www.gradeworkinggroup.org/), or other systems that provide a structured approach for assessing plausible causation that can be used with epidemiological evidence.

As in all scientific disciplines, research using FF&PC systems should strive for the highest standards of methodological rigor. The need for robust evidence has been emphasized in some recent reviews, which noted the evidence base linking ultra-processed food consumption to health outcomes was suboptimal and at high risk of bias [27,54]. The 2025–2030 United States Dietary Guidelines Advisory Committee (DGAC) emphasized the need for stronger evidence in its systematic review of the relationship between ultra-processed food consumption and growth, body composition, and risk of obesity [54]. Depending on the age and life-stage subgroup, the DGAC was either unable to draw conclusions because of evidence insufficiency or graded the overall strength of the evidence as limited, citing concerns over its inconsistency and lack of directness [54]. Similarly, in 2025, the United Kingdom Scientific Advisory Committee on Nutrition (SACN) highlighted limitations in the evidence base, noting difficulties in determining the extent to which observed associations between ultra-processed foods and adverse health outcomes could be explained through other established dietary relationships [27].

Furthermore, some of the available research has been criticized for not adequately controlling for potential confounding factors (e.g. macronutrient content, energy density, socioeconomic factors, lifestyle and dietary patterns, reporting, and misclassification bias [48,51,61]) or including inappropriate comparison groups [13]. Principle 3 is important because drawing definitive conclusions about health impacts without robust evidence can overstate the strength of the findings [51,57]. In cases where decision-making is time-sensitive, transparency and documentation regarding the data available and leveraged, as well as gaps, are critical. Researchers should therefore consider the implications of developing FF&PC systems based on preliminary evidence that may nevertheless be adopted by decision-makers or policymakers [48].

#### The impact that processing steps have on the final composition and structure of the food in terms of a putative effect on a health-related endpoint should be considered

The effects of processing steps, such as cooling, heating, freezing, mixing, extruding, filtering, cooking, fermenting, drying, forming, and packaging, can have positive, negative, or neutral impacts on a food’s physical, biological, or chemical properties, depending on the context. However, some FF&PC systems have been criticized for simplistically implying that food processing is inherently harmful [10,50,62]. Rather than relying on broad categorizations such as “highly processed” or “unprocessed,” principle 4 emphasizes that FF&PC systems should differentiate foods based on the specific processing steps involved and the evidence-based effects of those steps on final food properties and subsequent health-related endpoints (principle 2). A putative effect on a target health-related endpoint should be established to support any claim that the classification of foods based on processing is related to negative health outcomes. A demonstrated association, or at a minimum, a plausible hypothesis, is required to support the assumption that processing steps make foods more harmful or beneficial to human health. Researchers should specify the processing steps considered in the classification system and justify their inclusion based on demonstrated relationships with health-related endpoints.

To further avoid oversimplification, researchers should consider the nature of the food being processed, the interplay between multiple operations, and that both beneficial and adverse effects can result from the same processing step. Examples reflecting these nuances include extrusion used to make foods with different textures and different nutrient profiles (e.g. fiber-rich cereal, puffed snacks) [37], canned and frozen yellow corn retaining comparable nutrient profiles despite differences in processing steps [63], heating to reduce microbial risk whereas simultaneously degrading thermolabile nutrients (e.g. vitamin C) [64], treating milk with ultrahigh temperature to extend shelf-life while simultaneously altering protein structures in ways that affect digestibility [65], and cold-pressed juicing to preserve nutrients longer under refrigeration than using centrifugal methods [37,[63], [64], [65], [66]]. Ongoing advances in processing science and novel technologies further amplify this complexity [67,68], highlighting the need for FF&PC systems to remain dynamic and responsive to emerging innovations and understanding (principle 6).

Given the variable and context-specific effects of processing steps, researchers face the complex task of disentangling these relationships to generate sufficient evidence on how specific processing steps may influence health outcomes. It is, therefore, integral that FF&PC systems clearly distinguish between well-supported findings and areas of scientific uncertainty (principle 3). This challenge is compounded by the reality that identifying the processing steps a food has undergone may require specialized food science expertise, access to proprietary manufacturing information, or comprehensive processing databases—resources that are often limited or unavailable [38]. It is worth emphasizing that, beyond the identification of the industrial equipment, technological processes, and physical parameters involved in each of the processing steps, researchers can incorporate the extent to which each of these steps affects food structure and composition and subsequently encode food functionality and its impact on health outcomes [33]. In the absence of such knowledge and data, formulation (described in principle 5) should not be used as a proxy for the range or impact of processing steps, as they are 2 distinct concepts (principle 1) that should be individually assessed for their impact on food properties. When both formulation and processing are applied together in the design and production of a food, the impact on final food properties may be interdependent. As an example, nitrite (part of formulation) reactivity may be enhanced at high temperatures (part of processing).

#### The impact of formulation on the final composition and structure of the food in terms of a putative effect on a health-related endpoint should be considered

Principle 5 highlights that food formulations can have distinct and measurable impacts on a food depending on what is added, removed, or modified. This principle emphasizes that FF&PC systems should differentiate foods according to specific formulation components and their evidence-based effects on final food properties and health-related endpoints (principle 2), with considerations for the quantity and interplay between food components.

Formulation has been conflated with processing in some food classification systems, yet it is a distinct concept as discussed in principle 1 [10,11]. For instance, common components of formulation, such as the presence of additives intended for preservation (e.g. antioxidants), food safety (e.g. antimicrobials), modifying sensory features (e.g. flavors, colors), or technological properties (e.g. emulsifiers, gelling agents), have been used as markers to identify highly or ultra-processed foods that are presumed to be potentially harmful [8,69]. Such assumptions oversimplify the complex role of food components and fail to account for functional distinctions and the diversity of modern food supplies [43]. Distinguishing between processing and formulation enables a clearer understanding of how each independently influences food properties and health outcomes.

Like in principle 4, researchers should justify with evidence and clearly specify which aspects of formulation, if any, are considered in their classification system, their source of formulation information (e.g. ingredient lists, food composition or product databases, manufacturer data, patent filings, biochemical or laboratory analyses), the implications of relying on alternative data sources, and acknowledge any associated limitations and assumptions of their decisions in this regard (principle 1).

#### Systems should evolve over time to reflect advancements in science and changes in the food supply, with previous versions of a system being distinguishable from updated versions

FF&PC systems should be designed with built-in mechanisms to evolve in response to advances in science, shifts in food supplies, and emerging public health priorities, thereby avoiding obsolescence and misalignment with current understandings and limiting their utility for research and policy. Researchers must also decide whether a system requires minor refinements or fundamental changes to continue fulfilling its intended purpose or if complete abandonment is necessary.

A key shortcoming mentioned in the literature is a lack of transparent version control [41]. Critiques of the Nova system have highlighted its evolution, which has lacked systematic documentation of changes, undermining reproducibility and interpretability with undifferentiated iterations [26,41]. In line with principle 1, researchers should establish clear versioning protocols, document modifications, and explain the rationale for updates, assess the impact on reproducibility and interpretation, and minimize the creation of excessive exceptions. As an example, the Food Compass nutrient profiling system, which incorporates the Nova system to identify processing levels, has released clearly versioned updates informed by emerging evidence, new data, and community feedback, demonstrating how structured evolution can improve clarity and reproducibility while preserving the tool’s core purpose [70,71].

#### Current scientific evaluations from scientific bodies with relevant expertise should be consulted for each iteration

Principle 7 calls for researchers to acknowledge and consider core concepts from nutrition science and public health when developing and refining FF&PC systems, including the well-established benefits of including fruits, vegetables, pulses, and whole grains and limiting saturated and trans fats, sodium, and added sugars in the diet [72,73]. Researchers should explicitly consider existing evaluations by scientific bodies with relevant expertise. The outputs from institutions that synthesize large bodies of evidence to inform food safety standards, nutrient intake recommendations, dietary guidance, and regulation of additives and other ingredients may be consulted, including, but not limited to, the United States DGAC, United States Food and Drug Administration, USDA, United Kingdom SACN, European Food Safety Authority, FAO/WHO, and Codex Alimentarius.

Operationalizing a classification system that contradicts scientific consensus raises legitimate concerns about its validity and regulatory coherence and risks undermining public trust [74]. For example, items such as whole-grain bread, plant-based milk, or fortified cereal may be classified as highly or ultra-processed, potentially discouraging the consumption of foods that have been recommended as part of dietary guidelines [11,13,38]. This discrepancy is further illustrated by a modeling study showing that a diet primarily composed of ultra-processed foods as defined by the Nova system can be designed to meet most nutrient requirements and receive a high diet quality score [75]. Such inconsistencies highlight the need for careful system design.

Importantly, this principle does not require researchers to uncritically align with outputs from relevant scientific bodies but instead encourages them to consider these inputs in their development and refinement. To uphold transparency (principle 1), researchers should explicitly state whether and how extant evaluations from scientific bodies were incorporated, justify and document any divergences, and ensure classification and operational logic are not contradicted by widely accepted scientific consensus unless supported by robust evidence.

#### The context(s) in which a system was validated should be considered in its application

At its core, principle 8 ensures that FF&PC systems are scientifically valid, appropriately contextualized, and responsibly applied. To ensure this, FF&PC systems should be validated within their intended context before being applied more broadly, and unvalidated systems should be treated as exploratory. At least 2 types of validity apply. First, any instrument meant to assess FF&PC should be validated in the sense that the assessment measures what was actually consumed; such best practices have been outlined for the validation of dietary intake assessment tools (related to principle 1) [76]. In addition, the proposed principles are for FF&PC systems designed for health, so criterion validity for specified health outcomes should also be assessed (related to principles 2 and 3) [76].

No food classification system is universally applicable; each system is developed within a specific context, defined by target population, intended purpose, available datasets, and regulatory, regional, and cultural settings, and may not translate effectively across all use cases [39]. Understanding and respecting these contextual boundaries is essential to reducing misclassification and misinterpretation. Researchers should clearly define the scope and boundaries of their classification system and detail the original validation context, including the constraints, assumptions, and necessary conditions for generalization. Researchers applying an existing classification system outside its original validation context should report any modifications made and expressly acknowledge any potential limitations (principle 1).

#### The probative value of a research question or proposed FF&PC system should be considered before engaging in analysis or development

Developing or applying a classification system should be driven by a clear scientific purpose. Principle 9 calls on researchers to identify the specific gap or challenge their classification system or study is designed to address, articulate how it advances or improves on existing approaches, and clarify what new evidence, interpretation, or practical application it is intended to generate. In contrast, systems that closely replicate existing models without meaningful differentiation may contribute little conceptual advancement and risk adding redundancy to the literature. Although replication strengthens scientific rigor, repeating studies without a clear rationale can lead to superfluity and wasted resources [74]. Given the resource-intensive nature of developing, analyzing, or applying food classification systems, researchers should prioritize efforts with the greatest potential for meaningful impact.

This principle promotes intellectual discipline, encouraging researchers to provide a clear statement of the probative value of their work, engage with the existing research landscape, and justify their approach. Importantly, this principle does not discount the value of publishing completed studies as a safeguard against publication bias, but it underscores the importance of strategic design and clearly articulated probative value from the outset.

## Discussion

This perspective presents 9 guiding principles for the scientific community to consider when developing, refining, and applying FF&PC systems. Without endorsing or advocating for the creation or use of new or existing FF&PC systems, these principles provide a shared approach.

We anticipate that few existing FF&PC systems will fully align with all the principles. However, the intention of these principles is to support research and innovation, not to establish an insurmountable barrier. Indeed, the deliberate use of the word “should” in each principle, rather than “will,” “must,” or “shall,” is an indication of this spirit. Some of the principles, particularly those related to causal inference and mechanistic validation, are considered aspirational given the current state of evidence. As causal evidence and processing-focused databases emerge, these principles can be operationalized more fully. Any remaining evidence gaps can be used to identify future research priorities and areas for investment. When researchers choose to develop, refine, or apply an FF&PC system without meeting these principles (e.g. in the absence of strong foundational evidence), the onus should fall on the researcher to acknowledge limitations and explain the implications of such choices as described in principle 1.

Many of these principles reflect best practices in nutrition epidemiology that ideally would be embedded in any scientific inquiry from the outset. However, in practice, subjectivity enters at multiple stages of the research process, and rearticulating these concerns within subdomains of science is a valuable practice, particularly given the widespread criticisms of some FF&PC systems and their applications in the literature [10,11,13,26,[37], [38], [39], [40], [41], [42], [43], [44], [45], [46], [47], [48], [49], [50], [51], [52], [53]]. In the future, these principles could be formalized into a structured checklist, similar in spirit to CONSORT or PRISMA [77], to assist both system developers and users in evaluating them systematically. By following these principles, FF&PC systems can be developed and applied for a wide range of use cases, and they may be helpful for those implementing interventions or health policies, offering a framework for assessing whether the research behind FF&PC systems underpinning those efforts is fit for purpose.

These principles can complement recent and ongoing initiatives from a growing number of organizations that are actively engaging with the complexities of these FF&PC systems, although not all such efforts are listed here [47,78,79]. The British Nutrition Foundation’s position statement on the concept of ultra-processed foods echoes several of the principles outlined here [78]. It highlights the need to establish mechanistic links between food attributes or processing techniques and health outcomes, as well as to distinguish between types of processing methods [78]. It also acknowledges challenges with data availability in composition databases, as well as the need to research the potential effects of avoiding ultra-processed foods [78]. The International Union of Food Science and Technology (IUFoST) recently proposed the IUFoST Formulation and Processing Classification scheme, which aims to address ambiguities in existing FF&PC systems, such as Nova, by separately evaluating formulation and processing factors, offering quantitative tools to assess nutritional and health impacts and incorporate a host of relevant food attributes in product classification (e.g. safety, sustainability, palatability, affordability, convenience) [47]. The Novo Nordisk Foundation is funding a project led by researchers at the University of Copenhagen to build a science-based understanding of how food processing methods and additives impact health [79]. In addition, the USDA’s research roadmap identified 6 research questions focused on understanding how ultra-processed foods impact food intake and cardiometabolic disease [80]. Together, these efforts reflect a shared commitment to improving FF&PC systems and underscore the value of clear principles to guide their development.

In conclusion, these 9 principles are intended to support the development and application of FF&PC systems focused on processing and formulation that are transparent, reflect biological plausibility, and are capable of supporting meaningful interpretations and informed actions. They are aspirational yet actionable, flexible to evolving science, and agnostic to system design, allowing adaptability across different use cases. These principles set high standards for scientific rigor while also acknowledging the possible need for public health judgments in the face of incomplete evidence. To quote Albert Einstein, “For science can only ascertain what is, but not what should be, and outside of its domain value judgments of all kinds remain necessary.” As debates continue about the role of food processing in health, a principled approach can help the scientific community avoid conceptual drift and ultimately better serve public health.

## Author contributions

The authors’ responsibilities were as follows – JTB, AWB, BB-F, ME, JMH, PAH: received travel reimbursement to participate in the IAFNS Writing Team Meetings and Workshop on 16 January, 2025, and 15–16 April, 2025, in Washington, DC; JTB: received funding for preparation of the initial draft; and all authors: contributed to content design, writing, final content, and editing, read and approved the final manuscript.

## Funding

This work is supported by the USDA National Institute of Food and Agriculture, AFRI project 1033399 and the Institute for the Advancement of Food and Nutrition Sciences (IAFNS) Food Classification Working Group. IAFNS is a nonprofit science organization that pools funding from industry and advances science through the in-kind and financial contributions from private and public sector members. This article, in part, includes information from a cross-stakeholder “Workshop on science-based principles for food classification focused on processing and formulation to support public health,” held on 15 April, 2025, by IAFNS in Washington, DC (see: https://iafns.org/event/workshop-on-science-based-principles-for-food-classification/). This dialogue included presentations from academia, industry, trade associations, and Federal government employees.

## Conflict of interest

AWB reports a relationship with Soy Nutrition Institute Global that includes: consulting or advisory and travel reimbursement. AWB reports a relationship with Calorie Control Council that includes: speaking and lecture fees. AWB reports a relationship with Potatoes USA that includes: speaking and lecture fees. AWB reports a relationship with National Cattlemen’s Beef Association that includes: consulting or advisory. AWB reports a relationship with Alliance for Potato Research and Education that includes: funding grants. BB-F reports a relationship with McCormick and Company Inc that includes: travel reimbursement. BB-F reports a relationship with Nutrient Institute that includes: consulting or advisory. BB-F reports a relationship with NutriScience Innovation that includes: consulting or advisory. If there are other authors, they declare that they have no known competing financial interests or personal relationships that could have appeared to influence the work reported in this paper.

## References

[1] Hossain M.M., Uddin S.M.K., Sultana S., Wahab Y.A., Sagadevan S., Johan M.R. (2021). Authentication of halal and kosher meat and meat products: analytical approaches, current progresses and future prospects. Crit. Rev. Food Sci. Nutr..

[2] Poti J.M., Mendez M.A., Ng S.W., Popkin B.M. (2015). Is the degree of food processing and convenience linked with the nutritional quality of foods purchased by US households?. Am. J. Clin. Nutr..

[3] Monteiro C.A., Levy R.B., Claro R.M., Castro I.R., Cannon G. (2010). A new classification of foods based on the extent and purpose of their processing. Cad. Saude Publica..

[4] Eicher-Miller H.A., Fulgoni V.L., Keast D.R. (2012). Contributions of processed foods to dietary intake in the US from 2003–2008: a report of the food and nutrition science solutions joint task force of the Academy of Nutrition and Dietetics, American Society for Nutrition, Institute of Food Technologists, and International Food Information Council. J. Nutr..

[5] Asfaw A. (2011). Does consumption of processed foods explain disparities in the body weight of individuals? The case of Guatemala. Health Econ.

[6] Martinez-Steele E., Khandpur N., Batis C., Bes-Rastrollo M., Bonaccio M., Cediel G. (2023). Best practices for applying the Nova food classification system. Nat. Food..

[7] Slimani N., Deharveng G., Southgate D.A.T., Biessy C., Chajès V., van Bakel M.M.E. (2009). Contribution of highly industrially processed foods to the nutrient intakes and patterns of middle-aged populations in the European Prospective Investigation into Cancer and Nutrition Study. Eur. J. Clin. Nutr..

[8] Davidou S., Christodoulou A., Fardet A., Frank K. (2020). The holistico-reductionist Siga classification according to the degree of food processing: an evaluation of ultra-processed foods in French supermarkets. Food Funct..

[9] Institute of Food Technologists Get the Facts: Food Processing. https://www.ift.org/-/media/policy-advocacy/ift-comments/efsa/ift-food-processing-toolkit.pdf.

[10] Botelho R., Araújo W., Pineli L. (2018). Food formulation and not processing level: conceptual divergences between public health and food science and technology sectors. Crit. Rev. Food Sci. Nutr..

[11] Levine A.S., Ubbink J. (2023). Ultra-processed foods: processing vs. formulation, Obes. Sci. Pract..

[12] World Health Organization (2025). https://www.who.int/news-room/articles-detail/call-for-experts-to-develop-a-who-guideline-on-consumption-of-ultra-processed-foods#:%7E:text=Deadline%20for%20submission%3A%2015%20June%202025%26text=The%20World%20Health%20Organization%20(WHO,be%20completed%20and%20a.

[13] Lockyer S., Spiro A., Berry S., He J., Loth S., Martinez-Inchausti A. (2023). How do we differentiate not demonise–Is there a role for healthier processed foods in an age of food insecurity? Proceedings of a roundtable event, Nutr. Bulletin.

[14] Pan American Health Organization, Pan American Health Organization Nutrient Profile Model, 2016. Washington, DC. Available from: https://iris.paho.org/server/api/core/bitstreams/e5f2ea91-bf73-4553-bffd-d157231ff353/content.

[15] Knowledge4Policy (2025). https://knowledge4policy.ec.europa.eu/health/food-guidelines-table-23_en.

[16] Health Canada (2019). Canada’s Dietary Guidelines.

[17] Ministry of Health of Brazil (2015). https://bvsms.saude.gov.br/bvs/publicacoes/dietary_guidelines_brazilian_population.pdf.

[18] Uruguay Ministry of Public Health (2016). Food-based Dietary Guidelines Uruguay.

[19] Food and Agriculture Organization of the United Nations (2019). https://www.fao.org/nutrition/education/food-dietary-guidelines/regions/countries/peru/en/.

[20] Israeli Ministry of Health (2020). Dietary Guidelines.

[21] Chile Ministry of Health (2022). Dietary Guidelines for Chile.

[22] Ministry of Public Health (2020). Food-based dietary guidelines - Ecuador.

[23] Koios D., Machado P., Lacy-Nichols J. (2022). Representations of ultra-processed foods: a global analysis of how dietary guidelines refer to levels of food processing. Int. J. Health Pol. Manag..

[24] González-Castell D., González-Cossío T., Barquera S., Rivera J.A. (2007). Contribution of processed foods to the energy, macronutrient and fiber intakes of Mexican children aged 1 to 4 years, Salud Publica Mex.

[25] Monteiro C.A., Cannon G., Levy R., Moubarac J.-C., Jaime P., Martins A.P. (2016). NOVA. The star shines bright. World Nutr.

[26] Sadler C.R., Grassby T., Hart K., Raats M., Sokolović M., Timotijevic L. (2021). Processed food classification: conceptualisation and challenges. Trends Food Sci. Technol..

[27] Scientific Advisory Committee on Nutrition, Processed foods and health: SACN’s rapid evidence update, 2025. Available from: https://assets.publishing.service.gov.uk/media/67ea98e4ba01abac8e9fe963/sacn-processed-foods-review.pdf.

[28] Monteiro C.A. (2009). Nutrition and health. The issue is not food, nor nutrients, so much as processing. Public Health Nutr.

[29] Srour B., Fezeu L.K., Kesse-Guyot E., Allès B., Méjean C., Andrianasolo R.M. (2019). Ultra-processed food intake and risk of cardiovascular disease: prospective cohort study (NutriNet-Santé). BMJ.

[30] Rico-Campà A., Martínez-González M.A., Alvarez-Alvarez I., de Deus Mendonça R., De La Fuente-Arrillaga C., Gómez-Donoso C. (2019). Association between consumption of ultra-processed foods and all cause mortality: SUN prospective cohort study. BMJ.

[31] Hall K.D., Ayuketah A., Brychta R., Cai H., Cassimatis T., Chen K.Y. (2019). Ultra-processed diets cause excess calorie intake and weight gain: an inpatient randomized controlled trial of Ad Libitum food intake. Cell Metab..

[32] 2025 Dietary Guidelines Advisory Committee (2022). https://www.dietaryguidelines.gov/sites/default/files/2022-07/Proposed%20Scientific%20Questions_508c_Final.pdf.

[33] 2025 Dietary Guidelines Advisory Committee. 2024. Scientific Report of the 2025 Dietary Guidelines Advisory Committee: Advisory Report to the Secretary of Health and Human Services and Secretary of Agriculture. U.S. Department of Health and Human Services. Available from: 10.52570/DGAC2025

[34] Dicken S.J., Jassil F.C., Brown A., Kalis M., Stanley C., Ranson C. (2025). Ultraprocessed or minimally processed diets following healthy dietary guidelines on weight and cardiometabolic health: a randomized, crossover trial. Nat. Med..

[35] Food and Drug Administration, U.S. Department of Health and Human Services, U.S. Department of Agriculture. Ultra-Processed Foods; Request for Information; , July 23, 2025. Available from: https://www.federalregister.gov/documents/2025/07/25/2025-14089/ultra-processed-foods-request-for-information.

[36] California Legislature, AB-1264 Pupil nutrition: restricted school foods and ultraprocessed foods of concern: prohibition, 2025. Available from: https://leginfo.legislature.ca.gov/faces/billNavClient.xhtml?bill_id=202520260AB1264.

[37] Visioli F., Marangoni F., Fogliano V., Del Rio D., Martinez J.A., Kuhnle G. (2022). The ultra-processed foods hypothesis: a product processed well beyond the basic ingredients in the package. Nutr. Res. Rev.

[38] O’Connor L.E., Herrick K.A., Papier K. (2024). Handle with care: challenges associated with ultra-processed foods research. Int. J. Epidemiol..

[39] Moubarac J.-C., Parra D.C., Cannon G., Monteiro C.A. (2014). Food classification systems based on food processing: significance and implications for policies and actions: a systematic literature review and assessment. Curr. Obes. Rep..

[40] Trumbo P.R., Bleiweiss-Sande R., Campbell J.K., Decker E., Drewnowski A., Erdman J.W. (2024). Toward a science-based classification of processed foods to support meaningful research and effective health policies. Front. Nutr..

[41] Gibney M.J. (2018). Ultra-processed foods: definitions and policy issues. Curr. Dev. Nutr..

[42] Gibney M.J. (2022). Ultra-processed foods in public health nutrition: the unanswered questions. Br. J. Nutr..

[43] Azeredo H., Azeredo E. (2022). Ultraprocessed foods: bad nutrition or bad definition?. ACS Food Sci. Technol..

[44] Panahi S., Jones W., Duncan A.M., Ferland G., Keller H., Grantham A. (2022). Guidance and perspectives on highly processed foods. Appl. Physiol. Nutr. Metab..

[45] Jones J.M. (2019). Food processing: criteria for dietary guidance and public health?. Proc. Nutr. Soc..

[46] Forde C.G. (2023). Beyond ultra-processed: considering the future role of food processing in human health. Proc. Nutr. Soc..

[47] Ahrné L., Chen H., Henry C.J., Kim H.-S., Schneeman B., Windhab E.J. (2025). Defining the role of processing in food classification systems—the IUFoST formulation & processing approach. NPJ Sci. Food..

[48] Monteiro C.A., Astrup A. (2022). Does the concept of “ultra-processed foods” help inform dietary guidelines, beyond conventional classification systems? YES. Am. J. Clin. Nutr..

[49] Astrup A., Monteiro C. (2022). Does the concept of “ultra-processed foods” help inform dietary guidelines, beyond conventional classification systems? NO. Am. J. Clin. Nutr..

[50] Gibney M.J., Forde C.G. (2022). Nutrition research challenges for processed food and health. Nat. Food..

[51] Visioli F., Del Rio D., Fogliano V., Marangoni F., Ricci C., Poli A. (2025). Ultra-processed foods and health: are we correctly interpreting the available evidence?. Eur. J. Clin. Nutr..

[52] Gibney M.J., Forde C.G., Mullally D., Gibney E.R. (2017). Ultra-processed foods in human health: a critical appraisal. Am. J. Clin. Nutr..

[53] Forde C.G., Decker E.A. (2022). The importance of food processing and eating behavior in promoting healthy and sustainable diets. Annu. Rev. Nutr..

[54] F.C. Stanford, C. Taylor, D.M. Hoelscher, C.A.M. Anderson, S. Booth, A. Deierlein, et al., Dietary patterns with ultra-processed foods and growth, body composition, and risk of obesity: a systematic review. November 2024. U.S. Department of Agriculture, Food and Nutrition Service, Center for Nutrition Policy and Promotion, Nutrition Evidence Systematic Review. Available from: https://nesr.usda.gov/2025-dietary-guidelines-advisory-committee-systematic-reviews/dietary-patternsultraprocessed_growth-obesity. 39817921

[55] Principle n. (2025). Oxford English Dictionary.

[56] National Academies of Sciences, Engineering, and Medicine, Reproducibility and replicability in science, National Academies Press, Washington, DC 2019. 31596559

[57] Valicente V.M., Peng C.-H., Pacheco K.N., Lin L., Kielb E.I., Dawoodani E. (2023). Ultraprocessed foods and obesity risk: a critical review of reported mechanisms. Adv. Nutr..

[58] Brown A.W., Aslibekyan S., Bier D., Ferreira da Silva R., Hoover A., Klurfeld D.M. (2023). Toward more rigorous and informative nutritional epidemiology: the rational space between dismissal and defense of the status quo. Crit. Rev. Food Sci. Nutr..

[59] Lash Timothy L., VanderWeele Tyler J., Sebastien H., Rothman Kenneth J. (2021).

[60] Hill A.B. (1965). The environment and disease: association or causation?. Proc. R Soc. Med..

[61] Barbaresko J., Broeder J., Conrad J., Szczerba E., Lang A., Schlesinger S. (2025). Ultra-processed food consumption and human health: an umbrella review of systematic reviews with meta-analyses. Crit. Rev. Food Sci. Nutr..

[62] van Boekel M., Fogliano V., Pellegrini N., Stanton C., Scholz G., Lalljie S. (2010). A review on the beneficial aspects of food processing. Mol. Nutr. Food Res..

[63] Miller S.R., Knudson W.A. (2014). Nutrition and cost comparisons of select canned, frozen, and fresh fruits and vegetables. Am. J. Lifestyle Med..

[64] Khatun M.R., Khatun M.K., Islam M.S., Al-Reza S.M. (2019). Effect of different cooking methods on vitamin C content of some selected vegetables. Int. J. Curr. Microbiol. Appl. Sci..

[65] Krishna T.C., Najda A., Bains A., Tosif M.M., Papliński R., Kapłan M. (2021). Influence of ultra-heat treatment on properties of milk proteins. Polymers.

[66] Khaksar G., Assatarakul K., Sirikantaramas S. (2019). Effect of cold-pressed and normal centrifugal juicing on quality attributes of fresh juices: do cold-pressed juices harbor a superior nutritional quality and antioxidant capacity?. Heliyon.

[67] Hassoun A., Dankar I., Bhat Z., Bouzembrak Y. (2024). Unveiling the relationship between food unit operations and food industry 4.0: a short review. Heliyon.

[68] Knorr D., Sevenich R. (2023). Processed foods: from their emergence to resilient technologies. Compr. Rev. Food Sci. Food Safety..

[69] Monteiro C.A., Cannon G., Levy R.B., Moubarac J.-C., Louzada M.L.C., Rauber F. (2019). Ultra-processed foods: what they are and how to identify them. Public Health Nutr.

[70] Barrett E.M., Shi P., Blumberg J.B., O’Hearn M., Micha R., Mozaffarian D. (2024). Food Compass 2.0 is an improved nutrient profiling system to characterize healthfulness of foods and beverages. Nat. Food..

[71] Mozaffarian D., El-Abbadi N., O'Hearn M., Erndt-Marino J., Masters W., Jacques P. (2021). Food compass is a nutrient profiling system using expanded characteristics for assessing healthfulness of foods. Nat. Food..

[72] World Health Organization & Food and Agriculture Organization of the United Nations, What are healthy diets? Joint statement by the Food and Agriculture Organization of the United Nations and the World Health Organization, World Health Organization, 2024. Geneva. Available from: 10.4060/cd2223en.

[73] United States Department of Agriculture (2020). Dietary Guidelines for Americans 2020-2025.

[74] Shyam S., Salas-Salvadó J. (2025). The impact of investigator bias in nutrition research. Front. Nutr..

[75] Hess J.M., Comeau M.E., Casperson S., Slavin J.L., Johnson G.H., Messina M. (2023). Dietary guidelines meet NOVA: developing a menu for a healthy dietary pattern using ultra-processed foods. J. Nutr..

[76] Kirkpatrick S.I., Baranowski T., Subar A.F., Tooze J.A., Frongillo E.A. (2019). Best practices for conducting and interpreting studies to validate self-report dietary assessment methods. J. Acad. Nutr. Diet..

[77] Equator Network Enhancing the QUAlity and Transparency Of health Research. https://www.equator-network.org/.

[78] British Nutrition Foundation (2024). https://www.nutrition.org.uk/news/position-statement-on-the-concept-of-ultra-processed-foods-upf/.

[79] Nordisk Fonden Novo (2025). https://novonordiskfonden.dk/en/news/more-knowledge-better-health-how-nutritional-science-can-improve-peoples-lives-assist-the-green-transition-and-safeguard-the-healthcare-systems-of-the-future/.

[80] O’Connor L.E., Higgins K.A., Smiljanec K., Bergia R., Brown A.W., Baer D. (2023). Perspective: a research roadmap about ultra-processed foods and human health for the United States food system: proceedings from an interdisciplinary, multi-stakeholder workshop. Adv. Nutr..

