# Supplementary material for: Guiding Principles for Science-Based Food Classification Systems Focused on Processing and Formulation
Source: Adv Nutr. 2026 Jan 19;17(4):100577. doi: 10.1016/j.advnut.2025.100577 (PMC13100725; doi:10.1016/j.advnut.2025.100577)
Supplement: Multimedia component 1 [file mmc1.docx]

Perspective: Guiding Principles for Science-Based Food Classification Systems Focused on Processing and Formulation

Jodi T. Bernstein, PhD, RD, Jodi Bernstein Medical Writing, Toronto, ON, Canada

**Supplementary Material: Principles Development Method Detail**

*Development of the Task*

IAFNS conducted informational interviews with key stakeholders and groups to help shape the project concept and ensure it would be additive to the ongoing dialogue and needs of the scientific community. These interviews included nutrition scientists across sectors, professional organizations, and regulators. From this scan, the Principles concept was developed.

*Assembling a Writing Team*

The Working Group identified the expertise needed for Principles development, to be captured in a group of 6 to 8 public-sector individuals, namely: food science; nutrition research and methodology, including intervention studies; information evaluation and synthesis; dietary guidance; and sensory science. Experts were invited and informed of the task, the expected effort, and their individual roles. Specifically, the Writing Team was the lead on drafting the Principles and making decisions about what feedback would be incorporated from the broader community.

*Drafting the Principles*

To generate the first draft of the Principles, the following steps were undertaken:

- **Identification of the Purpose, Principles, Values, and Issues framework** (no reference available). Briefly, this framework allowed the Writing Team to identify key issues that needed to be addressed through Principles, with the understanding that users of the Principles may assign a different value to each statement depending upon the application.
- **Extraction of issues from the public domain.** A non-systematic literature and report review was conducted to identify issues and challenges with FF&PC systems from the peer-reviewed literature and other reports, such as that of the 2025-2030 US Dietary Guidelines Advisory Committee. A systematic literature review was not considered necessary because there was significant repetition of issues across these documents. The Writing Team therefore proceeded with a high degree of confidence that the main issues were captured.
- **Organization of issues into categories.** Once issues were captured, they were organized into a discreet set of categories with the idea that one Principle statement could address the category. Writing Team members were then assigned to each category.
- **In-person Writing Team meeting.** In January 2025, the Writing Team convened in Washington, DC to share their work on draft Principles for specific categories. Refinements were provided by other Writing Team members. Between January and April 2025, the drafts were refined for presentation at the public meeting.
- **Public Presentation of Principles drafts.** IAFNS organized a public meeting held on April 15, 2025 in which the Writing Team presented the draft Principles for feedback. The meeting was open to any interested stakeholder, and allowed both in-person and virtual participation. The Principles were provided in advance to registrants to promote constructive feedback.
- **Further Principles refinement and manuscript submission.** The Working Group and Writing Team convened on April 16, 2025 to review input from the public meeting and consider refinements. Over the following months, the manuscript was drafted and then submitted for peer-review.
